# Mogamulizumab for post-transplant relapse of adult T-cell leukemia/lymphoma: a case study

**DOI:** 10.1007/s12185-022-03447-0

**Published:** 2022-09-09

**Authors:** Makoto Hirosawa, Midori Goto, Masahiko Oku, Kenichi Akao, Noriaki Kitamura, Tsukasa Nakanishi, Aya Tanaka, Daisuke Niino, Takehiro Higashi, Hiroaki Morimoto, Junichi Tsukada

**Affiliations:** 1 Department of Hematology, University of Occupational and Environmental Health, 1-1 Iseigaoka, Yahatanishi-ku, Kitakyushu, 807-8556 Japan; 2 Department of Pathology and Cell Biology, School of Medicine, University of Occupational and Environmental Health, Kitakyushu, Japan

**Keywords:** Mogamulizumab, ATLL, GvHD, GvATLL

## Abstract

Mogamulizumab (MOG), a humanized monoclonal anti-CCR4 antibody, exerts strong antibody-dependent cellular cytotoxic effects on CCR4-positive adult T-cell leukemia/lymphoma (ATLL) cells. As CCR4 is highly expressed on regulatory T cells as well as ATLL cells, pre-transplant MOG induces severe graft-versus-host disease (GvHD). However, limited data are available on post-transplant use of MOG for relapsed ATLL. Here we describe the case of a patient with ATLL who experienced post-transplant relapse with involvement of peripheral blood, skin, lungs, and lymph nodes. Neither tacrolimus dose reduction nor cytotoxic chemotherapy was effective, but a single dose of MOG (1 mg/kg) induced complete remission. After treatment with MOG, leukemic cells in the peripheral blood rapidly disappeared, and the skin, lymph node, and lung lesions gradually regressed. Most notably, the long-term remission was accompanied by recurrence of moderate acute GvHD (grade II, skin stage 2, gut stage 1, liver stage 0). Our findings indicate that MOG can augment allogeneic immune-mediated anti-tumor reactions through graft-versus-ATLL (GvATLL) even during post-transplant relapse involving the lymph nodes and lungs, along with inducing GvHD.

## Introduction

Adult T-cell leukemia/lymphoma (ATLL) is a CD4-positive mature T-cell malignancy with a dismal prognosis due to its chemo-resistance and tendency to predispose patients to opportunistic pathogen infections [1–3]. Allogeneic hematopoietic stem cell transplantation (allo-HSCT) provides sustained long-term remission for patients with aggressive (unfavorable chronic, acute, and lymphoma type) ATLL. The prognosis of patients with ATLL has been improved through the introduction of allo-HSCT [4–6].

However, in addition to treatment-related mortality, the incidence of post-transplant relapse remains high at approximately 40% [5]. A nationwide retrospective study of 252 patients with post-transplant relapse of ATLL in Japan showed that the 2-year overall survival (OS) rate from the relapse date was 13.7%, with only 27 patients surviving for over 2 years after relapse [7]. A retrospective analysis of 35 patients with progression or relapse of ATLL after allo-HSCT in Nagasaki, Japan, reported that the median survival time after relapse or progression was 6.2 months [8].

The initial treatment for post-transplant relapse is tapering or withdrawal of immunosuppressive agents (ISAs). Dose reduction or withdrawal of ISAs induces allogeneic immune-mediated anti-tumor effects called graft-versus-ATLL (GvATLL), indicating the susceptibility of patients with ATLL to post-transplant allogeneic immune reactions [5, 9–12]. A retrospective study of patients with ATLL who underwent allo-HSCT revealed that when ISAs were tapered and discontinued in six patients with relapsed or residual ATLL, four of the patients achieved remission [9]. Fukushima et al. reported that among ten patients with post-transplant relapse of ATLL, three patients achieved a complete remission (CR) following dose reduction or withdrawal of ISAs [5]. However, salvage chemotherapy after relapse has no significant effects on OS. Treatment options for post-transplant relapse of ATLL are limited.

Mogamulizumab (MOG) is a humanized monoclonal anti-C–C chemokine receptor type 4 (CCR4) antibody, which has been demonstrated to be clinically effective for ATLL [13–15]. As CCR4 is highly expressed on regulatory T cells (Treg cells) and ATLL cells, pre-transplant MOG use induces severe GvHD. Pre-transplant use of MOG less than 50 days before transplantation is associated with an increased risk of corticosteroid-resistant severe acute GvHD (aGvHD) [16]. However, limited evidence is available regarding post-transplant use of MOG.

Here, we describe the case of a patient with post-transplant relapse of ATLL. The peripheral blood (PB), skin, lungs, and lymph nodes (LNs) were affected during the relapse. In our case, a single administration of MOG induced a durable CR. Further, long-term remission was accompanied by recurrence of aGvHD.

## Case

**Fig. 1:**
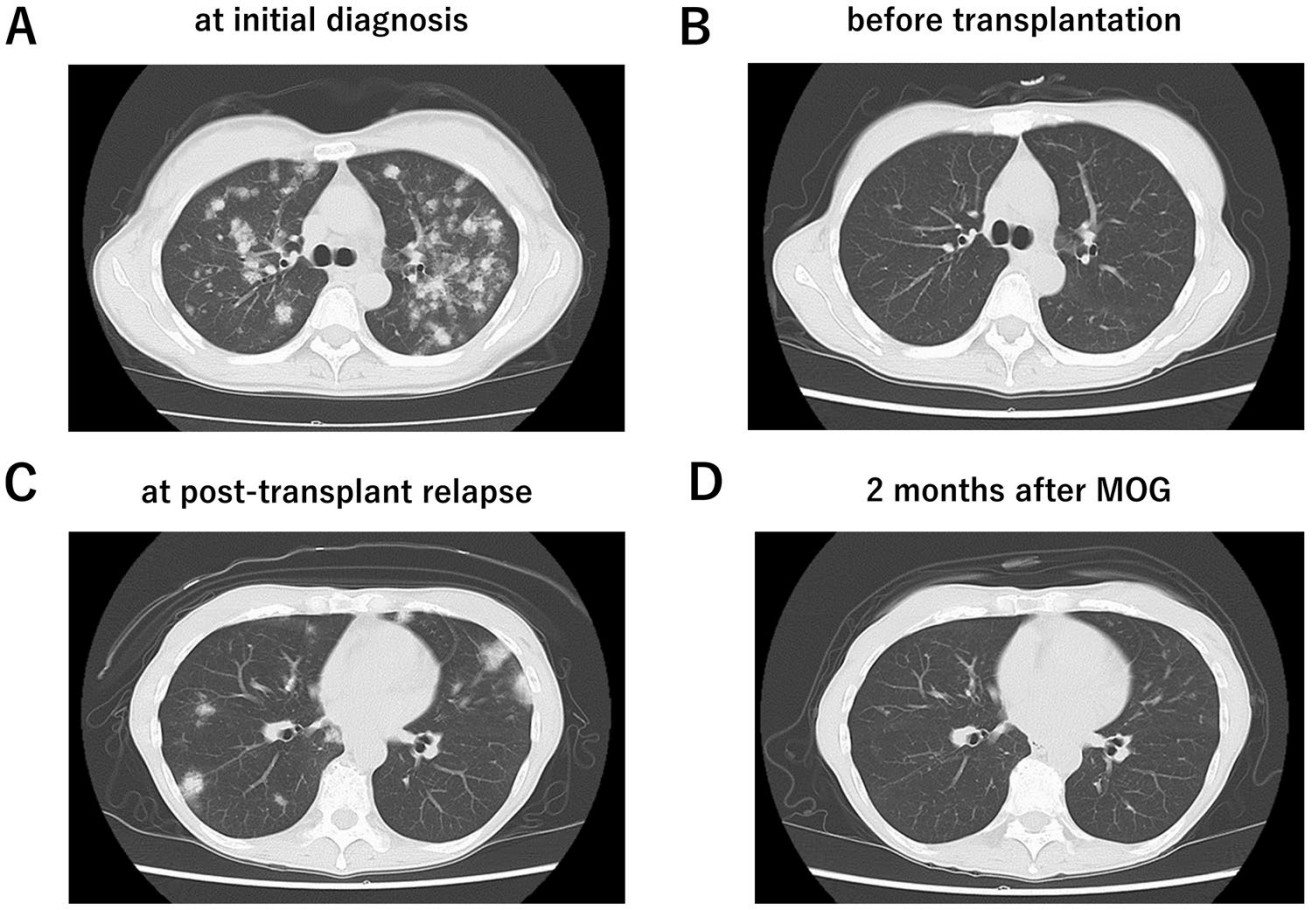
Computed tomography (CT) images of the chest at the initial diagnosis of adult T-cell leukemia/lymphoma (ATLL) (**A**), before bone marrow transplantation (**B**), during post-transplant relapse (**C**), and 2 months after mogamulizumab (MOG) therapy (**D**)

A 56-year-old Japanese female was referred to us for PB lymphocytosis with erythematous skin, lung, and LN lesions. The patient had no history of pulmonary diseases and was diagnosed with acute type ATLL, based on the PB, skin, and lung findings (Fig. 1A). Partial lobectomy was performed for the lung lesions. The lesions showed proliferation of atypical lymphoid cells, which were positive for CD3, CD4, and CD25 on flow cytometry (FCM) and immunohistochemistry (IHC). Monoclonal integration of human T-cell lymphotropic virus type 1 (HTLV-I) proviral DNA was confirmed with Southern blotting. After combination chemotherapy, she achieved remission (Fig. 1B) and received bone marrow transplantation (BMT) from a human leukocyte antigen-matched unrelated donor, with a reduced conditioning regimen of fludarabine 25 mg/m^2^ (day-7 to day-3), melphalan 80 mg/m^2^ (day-2), and total body irradiation 4 Gy (day-1). Tacrolimus and short-term methotrexate were used as GvHD prophylaxis. aGvHD grade I (skin stage 1, gut stage 0, and liver stage 0) developed following hematopoietic engraftment. Full donor chimerism was confirmed by variable number of tandem repeat analysis.

**Fig. 2:**
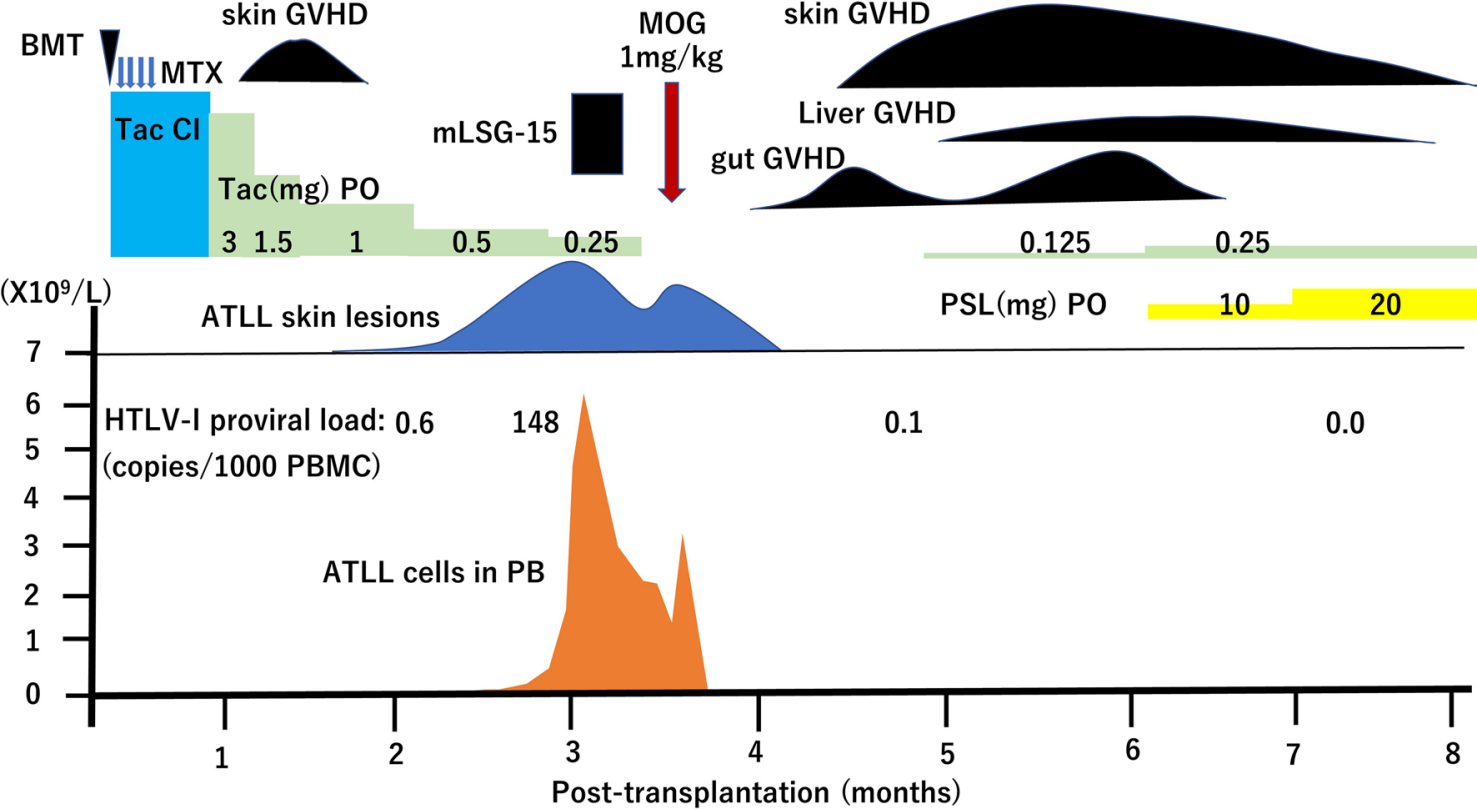
Clinical course. *BMT* bone marrow transplantation, *MTX* methotrexate, *Tac CI* tacrolimus continuous infusion, *Tac PO* tacrolimus oral administration, *ATLL* adult T-cell leukemia/lymphoma, *MOG* mogamulizumab, *PSL* prednisolone, *PBMC* peripheral blood mononuclear cell, *mLSG-15* mLSG-15 salvage chemotherapy

However, as shown in Fig. 2, 3 months after BMT, PB, skin, lung (Fig. 1C), and LN lesions recurred. The skin lesions revealed infiltration of atypical lymphoid cells positive for CD3, CD4, and CD25. In the epidermis, Pautrier-like microabscesses were formed by ATLL cells. The blood leukocyte count was 28.1 × 10^9^/L with 27% of abnormal lymphoid cells. The cells characterized morphologically by nuclear convolutions were positive for CCR4, CD3, CD4, and CD25. Chromosomal analysis using G-banding showed 45, X with loss of chromosome X. HTLV-I proviral load (HPL) increased to 148 copies/1000 PB mononuclear cells (PBMCs) with elevated levels of soluble interleukin-2 receptor (2761 U/mL). These findings were indicative of relapsed ATLL.

Although tacrolimus was tapered, no clinical response was obtained. The patient’s condition deteriorated even after mLSG-15 chemotherapy [17], with the development of progressive skin and LN lesions and a leukocyte count of 3.8 × 10^9^/L with 82% of abnormal cells.

Therefore, the patient received MOG (1 mg/kg) 105 days after BMT. Tacrolimus was discontinued. As a result, ATLL cells in the PB rapidly disappeared. The LN, lung (Fig. 1D), and skin lesions gradually regressed. HPL decreased to 0.1 copies/1000 PBMCs. As the patient experienced Epstein–Barr virus reactivation before MOG treatment and cytomegalovirus (CMV) antigenemia recurrence 5 days after MOG initiation, MOG was discontinued after one dose. CMV antigenemia was immediately treated with valganciclovir.

**Fig. 3:**
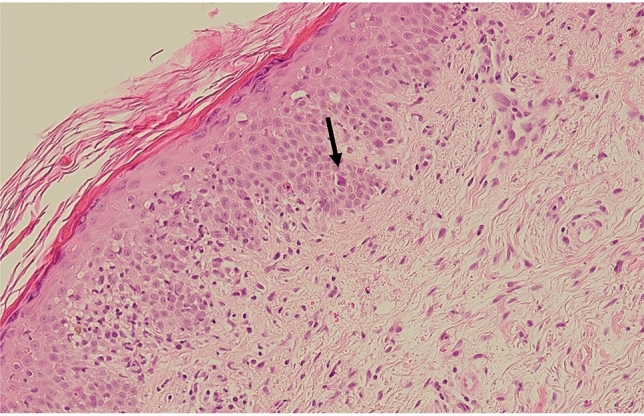
Hematoxylin and eosin-stained skin biopsy tissues performed during the recurrence of cutaneous acute GVHD after post-transplant mogamulizumab administration (original magnification × 100). The arrow shows an apoptotic cell

1 month after the initiation of MOG, signs of aGvHD grade II (skin stage 2, gut stage 1, and liver stage 0) developed in the skin, gut, and liver. GvHD was confirmed by skin biopsy (Fig. 3), which revealed mild vacuolar changes associated with apoptotic cells and lymphocytic inflammatory cells at the epidermal–dermal interface. Colonoscopic examination showed diffuse edema and patchy erosions. Serum levels of liver enzyme alanine aminotransferase were elevated. Prednisolone combined with a small dose of tacrolimus improved the GvHD manifestations. Using FCM, PB cell counts of CD3 + T cells, CD56 + CD3- natural killer (NK) cells, and CD4 + CD25 + CD127 − /low Treg cells were 921, 87, and 5/μL, respectively. HPL dropped to undetectable levels. The patient has been in remission for one year since the administration of MOG as an outpatient at our hospital. Tacrolimus was kept at low doses due to mild chronic GvHD (cGvHD) of the skin, eyes and liver. Long-lasting suppression of Treg cells was observed by FCM follow-up one year after MOG administration (CD3 + T cells, 1223/μL; CD56 + CD3- NK cells, 60/μL; CD4 + CD25 + CD127 − /low Treg cells, 11/μL).

**Fig. 4:**
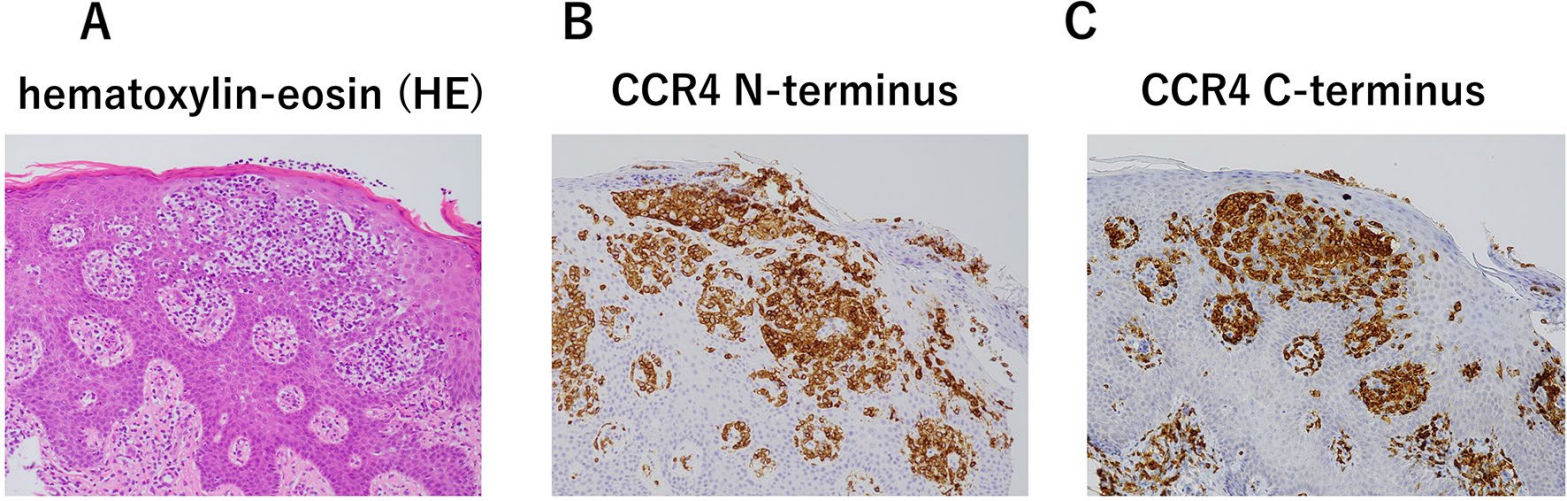
Expression of the C-terminus of CCR4 in skin lesions at post-transplant relapse. The skin lesions showed an infiltrate of medium to large-sized atypical lymphocytes (**A**, hematoxylin–eosin (HE) stain, original magnification × 100). The atypical cells formed Pautrier-like microabscesses and were immunohistochemically positive for the N-terminus of CCR4 (**B**, CCR4-N-IHC) and the C-terminus of CCR4 (**C**, CCR4-C-IHC)

Later, IHC for the N-terminus (CCR4-N-IHC) and C-terminus (CCR4-C-IHC) of CCR4 was performed, using antibodies against the N-terminus (POTELIGEO® TEST IHC, MINARIS Medical Cooperation, Tokyo, Japan) and C-terminus (amino acids 335–360: Novus Biologicals, CO, USA) of CCR4 [18] and skin biopsy samples obtained at post-transplant relapse. The tumor cells were strongly positive for CCR4-C-IHC as well as CCR4-N-IHC (Fig. 4).

## Discussion

GvATLL has been reported to develop in patients with ATLL who underwent allo-HSCT. Our findings suggest the development of GvATLL with MOG treatment in post-transplant relapse. The Nagasaki transplant group retrospectively analyzed 12 patients with post-transplant relapse of ATLL, who were treated with MOG or lenalidomide (LEN) [19]. In nine patients who received MOG, three patients achieved CR, and two of them remained in CR for more than 2 years. Progression or development of GvHD occurred in the LEN group but not in the MOG. In a retrospective analysis of six patients with post-transplant relapse of ATLL treated with MOG, Inoue et al. reported that, although ATLL cells in PB disappeared rapidly after MOG administration, LN lesions were resistant to MOG [20]. In their study, MOG was administrated more than 3 months after transplantation, and contrary to the high incidence of pre-transplant MOG-induced GvHD, neither progression nor emergence of GvHD was observed after MOG administration. Additionally, the development of grade II-IV aGvHD and cytopenia have been observed in patients with multiple myeloma received LEN maintenance treatment after allo-HSCT [21–23]. Therefore, our patient was treated with MOG.

In the present report, our patient experienced ATLL relapse involving the skin, lungs, LNs and PB, and received MOG 105 days post-transplantation. Although rapid disappearance of ATLL cells in the PB after MOG administration might be due to its direct anti-leukemic effect, the patient achieved long-term remission of skin, LN, and lung lesions and experienced a recurrence of aGvHD. These findings are supported by several studies. Tamai et al. reported that a patient with post-transplant relapse of ATLL achieved partial remission following the emergence of mild skin aGvHD during MOG therapy [24]. Moreover, a long-term CR was achieved in the presence of GvHD. A prospective study of patients with ATLL who underwent allo-HSCT revealed that grade I-II aGvHD was associated with a higher OS and progression-free survival than those of patients without GvHD [25]. A nationwide retrospective study in Japan including 294 patients with ATLL revealed the association of mild-to-moderate aGvHD with a lower risk of disease progression after allo-HSCT [12]. In addition, Ishida et al. showed a significant association of limited and extensive cGvHD with reduced ATLL-related mortality and a positive impact of extensive cGvHD on OS in a retrospective study of 616 patients with ATLL underwent allo-HSCT other than cord blood transplantation [26]. In a retrospective analysis of 35 ATLL patients with post-transplant relapse, three patients achieved a durable CR along with emergence or progression of cGvHD after donor leukocyte infusion (DLI) [8]. Our patient showed extensive cGvHD after MOG.

Gain-of-function mutations of *CCR4* affecting the C-terminal domain of the protein [27] have been reported to determine sensitivity to MOG in ATLL [28–30]. The mutations are either frameshift or nonsense and impair CCR4 internalization [27]. In this regard, Fujii et al. reported that CCR4-C-IHC using an antibody against the CCR4 C-terminus (amino acids 335–360) was useful in estimating *CCR4* mutations [18]. The positivity of CCR4-C-IHC were inversely correlated with the *CCR4* mutation status. In our patient, tumor cells were strongly positive for CCR4-C-IHC. No high-risk chromosome abnormalities associated with ATLL-related death after allo-HSCT, such as loss of chromosome 14 and structural breakpoints at 3p, 1q, 5q and 6q [31] were observed.

The possible toxicities of post-transplant administration of MOG should be considered in clinical settings. CMV reactivation has been observed in patients treated with post-transplant MOG [19], where the interval between the initiation of MOG and CMV reactivation was only 6 days. In our patient, CMV reactivation occurred 5 days after MOG administration; however, neither retinitis nor pneumonia was observed, as immediate valganciclovir treatment was initiated.

Skin lesions have been found to be susceptible to GvATLL. Kamimura et al. reported two cases of post-transplant skin relapse of ATLL, where the patients achieved a long-term CR for 8 and 9 years after DLI [32]. A nationwide survey of relapsed or refractory ATLL after allo-HSCT in Japan revealed that patients who developed skin lesions during post-transplant relapse had a longer OS after relapse than patients who did not [7].

Our findings indicate that MOG augments allogeneic immune anti-tumor reactions through GvATLL even during relapse involving LNs and lungs. This suggests that, when patients with post-transplant relapse are unable to achieve sufficient levels of GvATLL to eradicate tumor cells, blockade of Treg cells by MOG may lead to immune modulation to treat or prevent relapse without excessive toxicity. However, the number of post-transplant MOG cycles required to differentiate GvATLL from severe GvHD remains unclear. Further prospective studies on MOG treatment for post-transplant relapse are required to answer this question.

## Data Availability

The authors declare that data supporting the findings of this study are available within the article.
